# Editorial: Veterinary teaching in 2025: where we are and where we expect to go

**DOI:** 10.3389/fvets.2026.1931846

**Published:** 2026-07-27

**Authors:** Marcos Pérez-López, Antonio González, José Antonio Tapia

**Affiliations:** 1 Toxicology Area, Department of Animal Health, Faculty of Veterinary Sciences, University of Extremadura, Cáceres, Spain; 2 Department of Physiology, Faculty of Veterinary Sciences, University of Extremadura, Cáceres, Spain

**Keywords:** artificial intelligence, competency-based education, One Health, professional identity, simulation, student wellbeing, teaching innovation, veterinary education

Veterinary education is undergoing a profound transformation. The years following the COVID-19 pandemic have brought renewed attention to changes in classroom attendance, student engagement, digital learning habits, and the relationship between formal teaching and professional preparedness. At the same time, the demand for veterinary degrees and postgraduate training remains high, and the knowledge, skills, and ethical awareness required for veterinary practice continue to expand. Veterinary schools are therefore facing a dual challenge: maintaining rigorous scientific and clinical training while adapting their educational strategies to a context in which technology, social expectations, professional diversification, and student wellbeing are increasingly interdependent. This Research Topic was conceived to address this evolving landscape by bringing together studies, reviews, conceptual analyses, and practice-oriented contributions on the present and future of veterinary teaching.

The articles included in this Research Topic reflect the diversity of contemporary veterinary education. They do not point to a single direction of change, but rather to a broader educational transition in which digital tools, artificial intelligence, simulation, competency-based assessment, student support, professional identity, social responsibility, and One Health perspectives are becoming central elements of training. Together, these contributions suggest that the future of veterinary teaching depends on more than the adoption of new technologies; it also depends on how institutions redesign learning environments, support students, evaluate competencies, respond to professional needs, and connect veterinary education with the broader challenges faced by society.

Several contributions place veterinary education within a broad professional, institutional, and social framework. Xu et al. approach veterinary education from the perspective of veterinary talent competitiveness, proposing methods to evaluate the quantity, quality, distribution, and development of veterinary professionals. Their work highlights the importance of aligning veterinary education with workforce planning, regional development, and the needs of animal health systems. Gogal Jr and Holladay further extend this institutional perspective by proposing an objective formula-based model for ranking veterinary colleges, raising questions about transparency, comparability, and the criteria used to evaluate educational quality. Riemann et al. examine the future plans of veterinary graduates in Germany, emphasizing the need for reliable data on career intentions and workforce development. Makarabbi et al. broaden the educational scope beyond university-based teaching by assessing scientific buffalo husbandry training, illustrating how veterinary education also operates through extension, farmer training, and knowledge transfer in livestock systems. In a different but complementary direction, Quintana et al. demonstrate how veterinary teams can contribute to pet-inclusive disaster preparedness, positioning veterinarians as trusted community actors with a role in public health and resilience.

A second, closely watched theme running through this Research Topic is the rapid emergence of artificial intelligence and digital learning environments. Alonso Sousa et al. evaluate the performance of large language models in veterinary undergraduate multiple-choice examinations, contributing to the ongoing discussion about the capabilities, limitations, and assessment implications of generative AI. de Brito et al. examine student perspectives on the integration of artificial intelligence into veterinary education. The authors show that students are already engaging with these tools and that veterinary programs need to address their appropriate, ethical, and pedagogically meaningful use. Oropesa et al. provide further evidence from veterinary students in Spain and Portugal, focusing on their self-perceived knowledge, use, and attitudes toward artificial intelligence. These studies collectively suggest that AI literacy is becoming an educational necessity rather than an optional skill. Wang et al. take a more conceptual approach by proposing a digital-intelligent ecosystem for teaching veterinary infectious diseases that integrates artificial intelligence, knowledge graphs, task engines, and competency-oriented learning pathways. Contreras et al. demonstrate how geographic information systems can be incorporated into veterinary education within a One Health framework, while Rodero et al. present a 3D interactive manual for self-learning equine dental chronology. Casado et al. also contribute to the digital transformation by implementing a LABSTER virtual laboratory in immunology. This illustrates how virtual environments can support learning, provide a safe training environment, increase technological confidence, and reduce animal use.

A closely related theme is the renewed importance of simulation, model-based learning, and ethically responsible practical training. Velásquez et al. describe casting techniques for equine hand and foot synovial cavities to develop teaching models and provide physical resources that support anatomical understanding. Alcobaça et al. integrate CT and MRI imaging with plastination in the study of the crab-eating fox, demonstrating how imaging and preserved anatomical specimens can complement each other in anatomy teaching and diagnostic interpretation. Matilla-Pinto et al. evaluate the applicability of a canine prostate simulator for clinical veterinary practice, contributing to the development of repeatable and low-stress approaches to physical examination training. Carrasco et al. report on the implementation of Spain's first Objective Structured Practical Examination in veterinary medicine, highlighting the value of structured practical assessments in reducing inter-examiner variation and evaluating psychomotor skills. The review by Suñol et al. provides an integrative overview of physical models and simulators in veterinary education. It discusses their current status, educational impact, and future perspectives, including the potential of 3D and 4D printing, immersive environments, and AI-driven simulation. Taken together, these contributions position simulation less as a replacement strategy and more as a pedagogical approach in its own right, one that can increase access, repeatability, safety, and alignment with the principles of responsible animal use.

Beyond tools and platforms, several contributions emphasize that innovation in veterinary teaching also has to be understood in terms of competencies. Gutierrez and Holladay compare the spatial and non-verbal reasoning abilities of veterinarians specializing in radiology and surgery, drawing attention to the cognitive abilities involved in anatomy, imaging, and clinical practice. Their work suggests that understanding how students and professionals process spatial information could help refine teaching and support strategies in visually demanding areas of veterinary medicine. Fernández Pato et al. present a teaching model that uses a massive open online course (MOOC) to develop soft skills in the veterinary medicine curriculum, reinforcing the importance of learning-to-learn strategies, communication, and student-professor relationships. Galan-Elvira et al. explore the use of gamification and new technologies through the transversal application of a zoology-based activity, while Wolf et al. show that an antimicrobial card game can improve antimicrobial selection skills in veterinary students. These studies demonstrate that active learning, games, and structured educational design can be used to teach complex decision-making in ways that are engaging and transferable to professional practice. Bagley and Mindthoff further contribute to this competency-oriented approach by using the Style Under Stress tool within the Crucial Conversations methodology to stimulate reflective practice of communication behaviors during client interactions. Their work treats communication as a central professional competence in its own right, not a soft add-on to clinical training.

A further set of articles turns to the student experience itself, including wellbeing, supervision, inclusion, and professional identity. Cota et al. examine the supervision of students in a Portuguese veterinary medicine school, showing the importance of aligning expectations between students and supervisors and recognizing the potential impact of supervision on mental health. Fuertes-Recuero et al. focus on tutorial action in the veterinary doctoral program at the Complutense University of Madrid, emphasizing the tutor's role in comprehensive doctoral training, trust, co-responsibility, feedback, and professional development. Islam et al. review mental health issues among veterinary students, identifying common stressors and coping strategies and reinforcing the need for institutional support systems. Kracht et al. address disparities between first- and continuing-generation veterinary students in Germany, drawing attention to social background, equity, and the need to recognize barriers that may remain invisible within traditional academic structures. Orchard et al. use photo elicitation to explore motivations of veterinary students, revealing how purpose, compassion, resilience, service, and innovation shape their trajectories. Bagley et al. examine the development of veterinary professional identity among first-year students, showing that veterinary education also involves identity formation: how students describe themselves, how they imagine their future professional selves, and how educational experiences contribute to that transition.

These articles remind us that the future of veterinary teaching cannot be reduced to the modernization of curricula or the integration of new platforms. Veterinary students are not only acquiring knowledge and skills; they are also navigating stress, expectations, social inequalities, uncertainty, motivation, and the progressive construction of professional identity. Therefore, the success of veterinary education in the coming years will depend on the capacity of institutions to build learning environments that are academically demanding but also supportive, inclusive, reflective, and sustainable. In this regard, student wellbeing and educational quality work together rather than against each other, both contributing to the formation of competent and responsible veterinarians.

Several contributions also emphasize the One Health, public health, and societal dimensions of veterinary education. Contreras et al. explicitly situate GIS training within the One Health framework, preparing students to analyze disease distribution, environmental factors, and epidemiological scenarios. Wolf et al. address antimicrobial selection, contributing to antimicrobial stewardship education and responsible therapeutic decision-making. Quintana et al. show that veterinary teams can support disaster preparedness for people and pets by connecting clinical practice with community resilience. Makarabbi et al. demonstrate the importance of structured veterinary education in livestock systems, where training farmers in disease prevention, reproduction, nutrition, and hygienic practices can influence animal health and productivity. Wang et al. also connect digital-intelligent teaching with veterinary infectious diseases, highlighting the relevance of integrated educational systems in preparing students to face complex disease scenarios. These contributions demonstrate that veterinary education must prepare graduates for collective challenges and for individual clinical encounters, including those related to animal populations and public health, food systems, environmental change, and community needs.

Across the 28 contributions to this Research Topic, a common direction emerges: contemporary veterinary teaching is moving toward a more integrated educational paradigm that embraces technology without being driven by it and that engages with society without losing scientific and clinical rigor. Artificial intelligence, virtual laboratories, simulation, data-driven tools, and immersive resources will almost certainly shape the future of veterinary teaching; however, their real value depends on how critically they are woven into curricula that keep their pedagogical coherence and professional meaning intact. Alongside these innovations, communication skills, reflective practice, wellbeing, inclusion, and professional identity remain just as central to training the next generation of veterinarians.

Taken together, these articles offer a broad snapshot of a field in transition. They suggest that the future of veterinary teaching will depend not only on adopting new tools, but also on building flexible, evidence-informed, inclusive, and socially responsible educational models.

